# Trends and Gaps in Digital Precision Hypertension Management: Scoping Review

**DOI:** 10.2196/59841

**Published:** 2025-02-10

**Authors:** Namuun Clifford, Rachel Tunis, Adetimilehin Ariyo, Haoxiang Yu, Hyekyun Rhee, Kavita Radhakrishnan

**Affiliations:** 1 School of Nursing The University of Texas at Austin Austin, TX United States; 2 School of Information The University of Texas at Austin Austin, TX United States; 3 Department of Electrical and Computer Engineering The University of Texas at Austin Austin, TX United States

**Keywords:** precision health, hypertension, digital health, prediction models, personalization, phenotyping, machine learning, algorithms, mobile apps, mobile health

## Abstract

**Background:**

Hypertension (HTN) is the leading cause of cardiovascular disease morbidity and mortality worldwide. Despite effective treatments, most people with HTN do not have their blood pressure under control. Precision health strategies emphasizing predictive, preventive, and personalized care through digital tools offer notable opportunities to optimize the management of HTN.

**Objective:**

This scoping review aimed to fill a research gap in understanding the current state of precision health research using digital tools for the management of HTN in adults.

**Methods:**

This study used a scoping review framework to systematically search for articles in 5 databases published between 2013 and 2023. The included articles were thematically analyzed based on their precision health focus: personalized interventions, prediction models, and phenotyping. Data were extracted and summarized for study and sample characteristics, precision health focus, digital health technology, disciplines involved, and characteristics of personalized interventions.

**Results:**

After screening 883 articles, 46 were included; most studies had a precision health focus on personalized digital interventions (34/46, 74%), followed by prediction models (8/46, 17%) and phenotyping (4/46, 9%). Most studies (38/46, 82%) were conducted in or used data from North America or Europe, and 63% (29/46) of the studies came exclusively from the medical and health sciences, with 33% (15/46) of studies involving 2 or more disciplines. The most commonly used digital technologies were mobile phones (33/46, 72%), blood pressure monitors (18/46, 39%), and machine learning algorithms (11/46, 24%). In total, 45% (21/46) of the studies either did not report race or ethnicity data (14/46, 30%) or partially reported this information (7/46, 15%). For personalized intervention studies, nearly half (14/30, 47%) used 2 or less types of data for personalization, with only 7% (2/30) of the studies using social determinants of health data and no studies using physical environment or digital literacy data. Personalization characteristics of studies varied, with 43% (13/30) of studies using fully automated personalization approaches, 33% (10/30) using human-driven personalization, and 23% (7/30) using a hybrid approach.

**Conclusions:**

This scoping review provides a comprehensive mapping of the literature on the current trends and gaps in digital precision health research for the management of HTN in adults. Personalized digital interventions were the primary focus of most studies; however, the review highlighted the need for more precise definitions of *personalization* and the integration of more diverse data sources to improve the tailoring of interventions and promotion of health equity. In addition, there were significant gaps in the reporting of race and ethnicity data of participants, underuse of wearable devices for passive data collection, and the need for greater interdisciplinary collaboration to advance precision health research in digital HTN management.

**Trial Registration:**

OSF Registries osf.io/yuzf8; https://osf.io/yuzf8

## Introduction

### Background

Hypertension (HTN) is the leading preventable cause of cardiovascular disease and premature mortality worldwide, affecting an estimated 1.28 billion adults [1]. It surpasses smoking, diabetes, and obesity as the most significant modifiable risk factor, contributing to 54% of stroke and 47% of ischemic heart disease cases [2,3]. Despite mounting evidence that antihypertensive treatment can reduce morbidity and mortality, HTN remains underdiagnosed and undertreated [4,5]. Globally, nearly half of adults with HTN are unaware that they have the condition, and only 21% have their blood pressure (BP) under recommended levels [1]. Significant disparities exist in HTN prevalence and management. While 82% of individuals with HTN live in low- and middle-income countries, only 7.7% achieve BP control, compared to 28.4% in high-income countries [4,6]. These disparities have been linked to various social and environmental determinants that disproportionately affect individuals from racial and ethnic minority groups [7]. Given the considerable global health burden and inequities stemming from suboptimal HTN management, there is an urgent demand for innovative and scalable solutions for effective HTN prevention and control.

### Challenges in HTN Management

Despite the availability of effective treatments, only 21% of adults with HTN worldwide have their BP under control [6]. Longitudinal analyses have shown that up to 50% of patients stop taking their prescribed medications within 1 year; this is attributed to factors such as sociodemographics, medication side effects, lack of knowledge, comorbidities, lack of access to care, and patient-clinician relationship [8,9]. Current HTN treatment approaches are based on evidence from randomized controlled trials (RCTs) that reflect the mean results for the average patient [10]. This “one-size-fits-all” approach fails to consider the wide variation in an individual’s genetic, biological, behavioral, sociodemographic, and environmental factors that profoundly influence HTN treatment adherence and outcomes. This lack of personalization contributes to suboptimal treatment adherence and poor overall HTN control [9]. Effective management strategies must incorporate more nuanced approaches that adapt to the unique characteristics and contexts of individuals within diverse populations.

### Digital Precision Health for HTN Management

The emergence of precision health as a paradigm to empower individuals, predict and prevent disease before it starts, and personalize care addresses these challenges and presents a promising road map for transforming HTN management [11]. Expanding on precision medicine’s focus on personalized medical care, precision health goes beyond treatment, emphasizing health promotion and disease prevention for a more proactive approach to addressing population health [11-14]. Interventions in precision health are customized to the individual’s unique variations in genetic, biological, behavioral, sociocultural, and environmental determinants to improve health outcomes [14-16]. While personalized care has always been a goal in clinical practice, it is only with recent technological advancements in artificial intelligence, data analytics, and digital tools that the depth of personalization and predictive capabilities in disease risk or treatment response are becoming truly attainable [16]. Digital health technologies, including mobile apps, wearable devices, and remote monitoring, enable the harnessing of data to tailor interventions, predict disease risk, and engage patients more effectively in their care [11]. These tools present an opportunity to overcome traditional care barriers, facilitating more accessible, accurate, timely, and personalized HTN prevention and management strategies.

### Objectives

Although existing research has examined the effectiveness of digital interventions in improving health outcomes for HTN, there is a notable lack of prior reviews that integrate these findings in the context of precision health [12-15]. The primary objective of this scoping review is to synthesize the existing studies pertaining to precision health using various digital health technologies for the management of HTN in adults. Specifically, we aimed to address the following questions:

1. Which aspects of precision health are addressed (ie, prediction models, personalization, and phenotyping), and what are the characteristics of these studies?
2. Which digital health tools are being used in precision HTN management?
3. What are the characteristics of participants in precision health studies for HTN management?
4. For personalized interventions, what types of data are used for personalization, and what are the characteristics of personalization?

By mapping out the current state of digital precision health for HTN management, we seek to provide a foundation for future research, policy, and the development of effective and equitable interventions to improve the prevention, diagnosis, and management of HTN on a global scale.

## Methods

### Design

This scoping literature review was conducted following the framework for scoping reviews developed by Arksey and O’Malley [17]. This framework consists of five stages: (1) identifying the research question; (2) identifying relevant studies; (3) study selection; (4) charting the data; and (5) collating, summarizing, and reporting the results. Thematic analysis of findings were conducted based on the 3 categories of studies included in the review: personalized interventions, phenotyping, and prediction models. The review used the PRISMA-ScR (Preferred Reporting Items for Systematic Reviews and Meta-Analyses extension for Scoping Reviews) reporting guidelines to increase methodological transparency (Multimedia Appendix 1) [17]. The review protocol was registered with the OSF Registries (osf.io/yuzf8).

### Search Methods

In July 2023, assisted by a research librarian, we conducted comprehensive searches across 5 databases: PubMed, CINAHL, Web of Science, Embase, and Inspec. We filtered the search for peer-reviewed articles published between 2013 and 2023 in the English language. This 10-year time frame was chosen to capture the most recent and relevant advancements in digital health technologies and their application in HTN management. Search terms included “precision health” (eg, *precision medicine* OR *personalized* OR *tailored* OR *individualized*) AND “digital health” (*telemedicine* OR *telehealth* OR *mobile apps* OR *wearable electronic devices* OR *electronic health*) AND “hypertension” (*high blood pressure* OR *elevated blood pressure*). Medical subject headings terms were adapted across databases. The full search strategy is provided in Multimedia Appendix 2.

Final search results were transferred to an EndNote (version 20) database, and duplicates were removed. The remaining articles were imported into Covidence, a web-based software platform designed for conducting literature reviews. Blinded screening of title, abstract, and full texts was conducted by 3 authors (NC, RT, and AA), with each study independently reviewed by 2 of these authors. Any conflicts in opinion were reconciled through discussion or, if needed, by involving a third reviewer (HR).

### Eligibility Criteria

This scoping review sought to comprehensively map key concepts within the identified research area by including all types of research designs of original, peer-reviewed research papers. In addition, studies were included if they (1) sampled adults aged ≥18 years, (2) contained a diagnosis of HTN, (3) had a precision health focus, and (4) used a digital health technology. If studies included participants with >1 diagnosis (ie, HTN and diabetes mellitus), and HTN management was a primary focus of the intervention, they were included. To identify studies with a focus on precision health, if the term precision health or its synonyms (ie, personalized, tailored, individualized, predictive, and phenotype) were in the title, abstract, or main text, they were included. For the detailed eligibility criteria, refer to Textbox 1.

**Eligibility criteria for scoping review.**

**Inclusion criteria**

- Study type: original peer-reviewed research paper
- Period: studies published between January 1, 2013, and July 20, 2023
- Language: English
- Population: adults aged ≥18 years with a diagnosis of hypertension
- Had a precision health focus (ie, predictive, personalized, or phenotyping)
- Used a digital health technology (eg, mobile phones, telemedicine, wearable devices, or electronic health records)

**Exclusion criteria**

- Study types: case studies, editorials, opinion pieces, gray literature, dissertations, literature reviews, and trial protocols
- Period: studies published before January 1, 2013, or after July 20, 2023
- Language: any language other than English
- Population: pediatric or pregnant patients
- Pulmonary artery hypertension diagnosis
- Interventions targeting health care providers

Studies were excluded if they involved pediatric or pregnant participants, had a diagnosis of pulmonary artery HTN, or reported interventions targeting health care providers. Pregnant individuals were excluded due to the unique physiological and treatment differences in managing HTN during pregnancy, such as gestational HTN or preeclampsia, which is considerably outside the scope of this review. Furthermore, we excluded case studies, editorials, opinion pieces, gray literature, dissertations, literature reviews, and trial protocols, as these are secondary or nonempirical sources lacking original research data essential for our analysis.

### Data Extraction

We developed 3 separate data extraction templates, each containing fields for all identified key data elements. The first template was used to extract data on study characteristics, including study location, design, sample characteristics, precision health focus, digital health technology used, and disciplines involved. The other templates were used to extract data on personalization characteristics and types of data used for personalization of tailored intervention studies. For each included study, the first author (NC) and 1 of the 3 coauthors (RT, AA, and HY) independently extracted data using the standardized templates. This dual-reviewer approach was used to minimize errors and biases in the data extraction process. Any discrepancies between reviewers in the data extraction were resolved through discussion, with a third author available for consultation if consensus could not be reached.

### Data Synthesis

Included studies were categorized based on their precision health focus: personalization, phenotyping, and prediction models. We conducted thematic analysis to identify recurring themes and patterns across the studies, with themes derived both deductively from the research objectives and inductively from the study findings [18]. Studies were cross-compared to highlight differences and similarities in methodologies, designs, and outcomes. Synthesizing the extracted data allowed us to identify gaps in the current research landscape, generating recommendations for future research directions which we discuss in this study.

## Results

### Search Results

**Figure 1:**
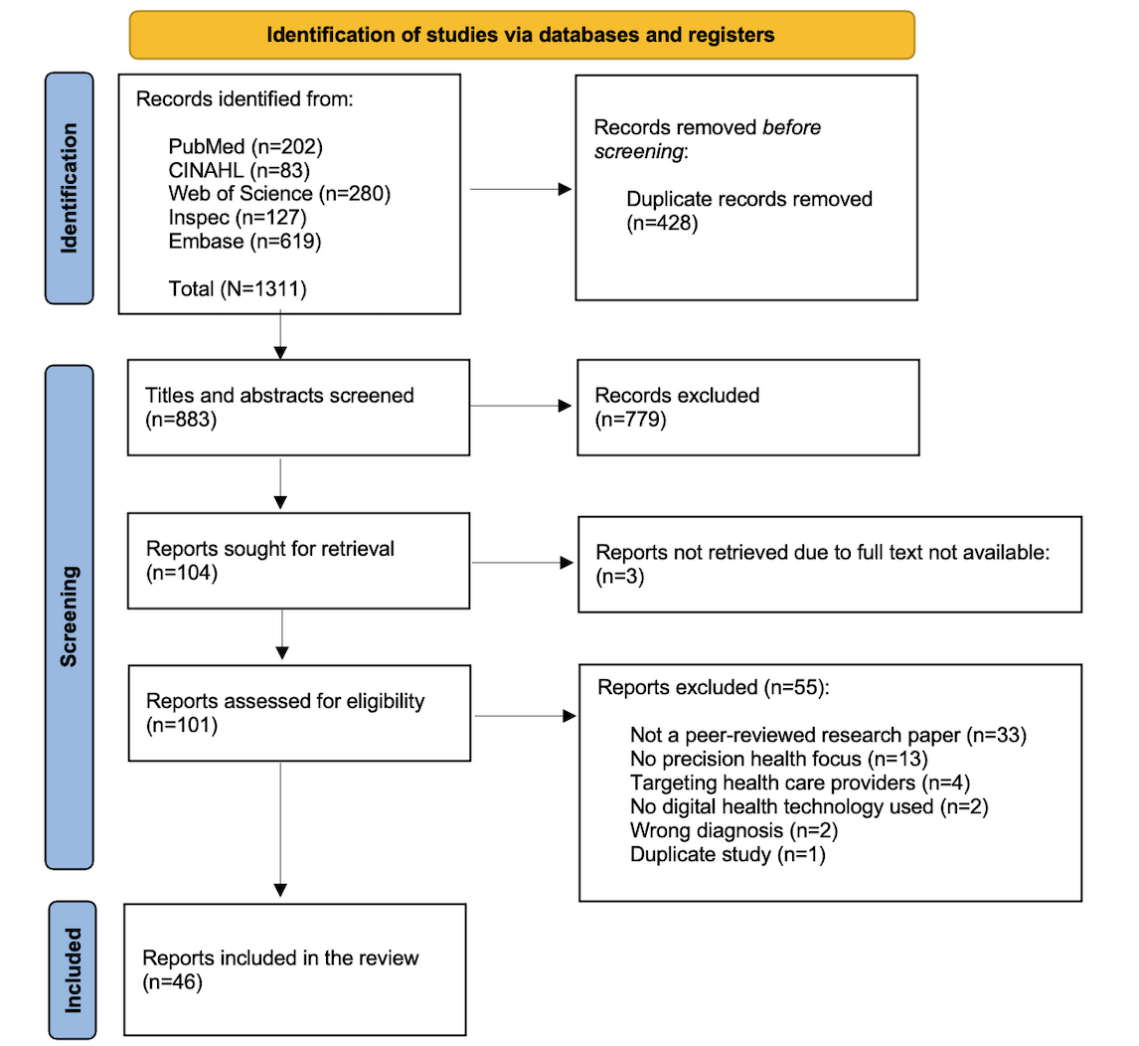
PRISMA (Preferred Reporting Items for Systematic Reviews and Meta-Analyses) flow diagram of study selection.

The search yielded 883 studies after the removal of duplicates; after title and abstract screening, we identified 104 (12%) studies for full-text review. Of the 104 studies, a final sample of 46 (44%) studies met the inclusion criteria for this review (Figure 1). In alignment with the scoping review methodology, we did not conduct a quality appraisal of the included studies, as our goal was to rapidly identify and synthesize the existing evidence of digital precision health research for HTN management [16].

### Summary of the Included Studies

Table 1 provides an overview of the included studies. Most studies were conducted in or used data from North America, predominantly from the United States (30/46, 65%), with 1 (2%) study conducted in Canada. Of the 46 studies, 7 (15%) were conducted in or used data from Europe, followed by 6 (13%) from Asia and 1 (2%) from Brazil in South America. Of the 46 studies, 1 (2%) study on genome sequencing used data from both the United States and the United Kingdom [19]. Nearly half of the studies (21/46, 46%) were RCTs, of which 8 (17%) were pilot or feasibility RCTs. There was a diverse mix of studies comprising qualitative, mixed methods, and analytical observational (eg, cohort and cross-sectional) designs in addition to the RCTs.

**Figure 2:**
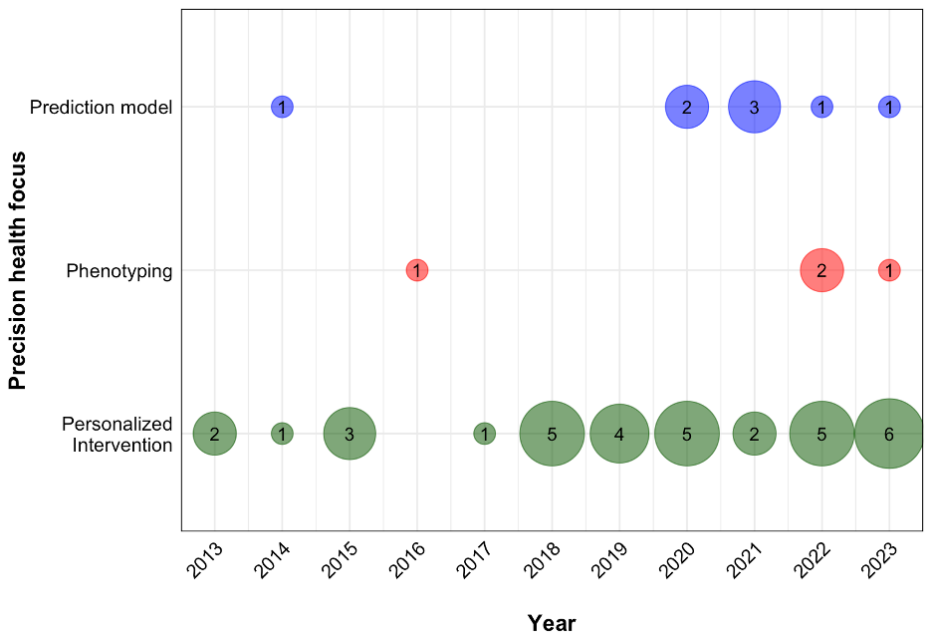
Studies published by year and precision health focus.

In terms of precision health focus, most studies (34/46, 74%) focused on personalization, 8 (17%) focused on prediction models, and 4 (9%) focused on phenotyping (Figure 2). Disciplines involved were predominantly within the health sciences (29/46, 63%), with over a third of studies (15/46, 33%) featuring interdisciplinary teams comprising ≥2 disciplines (eg, medical and health sciences, informatics, and computer and electrical engineering). All included studies (46/46, 100%) sampled populations with HTN, with additional diagnoses of diabetes mellitus (16/46, 35%), hyperlipidemia (7/46, 15%), and classifications of overweight or obesity (3/46, 7%).

**Table 1:** Summary of the included studies (N=46).

Characteristics and categories		Studies, n (%)
**Study location**		
	North America (the United States and Canada)	31 (67)
	Europe (Spain, Ireland, the United Kingdom, and Sweden)	7 (15)
	Asia (South Korea, Japan, Lebanon, Hong Kong, and China)	6 (13)
	South America (Brazil)	1 (2)
	Multiple locations (the United States and the United Kingdom)	1 (2)
**Study design**		
	RCTs^a^ (including pilot or feasibility)	21 (46)
	Analytical observational	16 (35)
	Qualitative	5 (11)
	Mixed methods	3 (7)
	Proof of concept	1 (2)
**Precision health focus**		
	Personalization	34 (74)
	Prediction models	8 (17)
	Phenotyping	4 (9)
**Disciplines involved**		
	Health sciences	29 (63)
	Computer and electrical engineering	1 (2)
	Informatics and communication sciences	1 (2)
	Interdisciplinary team (≥2 disciplines)	15 (33)
**Clinical conditions of the sample**		
	Hypertension	46 (100)
	Diabetes mellitus	16 (35)
	Hyperlipidemia	7 (15)
	Overweight or obesity	3 (7)
	Others (bipolar disorder, kidney transplant, and stroke)	3 (7)

^a^RCT: randomized controlled trial.

### Study Characteristics

Study characteristics are presented in Multimedia Appendix 3, and a summary of the sample characteristics is presented in Table 2. Sample sizes in the studies ranged from 7 to 764,135. Nearly half (21/46, 46%) of the studies had a sample size <100, followed by 11 (24%) studies with a sample size between 100 and 499 and 6 (13%) with a sample size >10,000. Most studies (33/46, 72%) included participants with a mean age between 50 and 69 years. The range of female participants across all studies was 1.4% to 100%, with a median of 51% (IQR 40-62). Of the 46 studies, 14 (30%) did not report the race or ethnicity of participants.

**Table 2:** Summary of sample characteristics from the included studies (N=46).

Characteristic and category			Studies
**Sample size, n (%)**			
	<99	21 (46)	
	100-499	11 (23.9)	
	500-999	3 (6.5)	
	1000-9999	5 (10.87)	
	>10,000	6 (13.04)	
**Mean age^a^ (y), n (%)**			
	18-29	0 (0)	
	30-39	0 (0)	
	40-49	5 (11)	
	50-59	18 (39)	
	60-69	15 (33)	
	>70	1 (2)	
	Not reported	2 (4)	
**Race and ethnicity^b^**			
	African American or Black, n (median %^c^, IQR)	21 (39.7, 11.8-53.8)	
	American Indian or Alaska Native, n (median %^c^, IQR)	0 (0, 0)	
	Asian, n (median %^c^, IQR)	7 (1.0, 0-2.8)	
	Hawaiian or Pacific Islander, n (median %^c^, IQR)	0 (0, 0)	
	Hispanic or Latinx, n (median %^c^, IQR)	17 (5.2, 2.1-14.3)	
	Non-Hispanic White, n (median %^c^, IQR)	23 (50.9, 23.0-74.6)	
	Other, n (median %^c^, IQR)	12 (2.8, 0-4.7)	
	Not reported, n	14	
	Partially reported, n	7	
**Gender^d^**			
	Female, n (median %, IQR)	39 (51, 40-62)	
	Not reported, n	2	

^a^Mean age: Studies that cited another study or database for participant age (n=6) were excluded from the breakdown above.

^b^Race and ethnicity: Studies that cited another study or database for race and ethnicity (n=4) were excluded from the breakdown above.

^c^Median %: median percentage across the reported studies.

^d^Gender: Studies that cited another study or database for gender (n=5) were excluded from the breakdown above.

### Digital Personalization for HTN Management

#### Overview

Of the 46 studies, 34 (74%) had a precision health focus of personalization in HTN management (Table 3). These studies tested or assessed digital interventions that were tailored to the individual or group characteristics of its users. Most studies (30/34, 88%) tested a personalized digital intervention and were primarily quantitative in design (eg, cohort, RCTs and mixed methods). Of the 34 personalization studies, 4 (13%) were qualitative and described participants’ views on technology-based interventions. Sample sizes ranged from 11 to 10,803, and the most commonly reported primary outcomes included BP (21/34, 62%), medication adherence (4/34, 12%), participants’ views or feedback (4/34, 12%), and feasibility and satisfaction of the intervention (3/34, 9%). Digital technologies used most often in these studies included mobile phones (30/34, 88%), followed by BP monitors (17/34, 50%), wearable devices (4/34, 12%), electronic medication trays or pillboxes (3/34, 9%), web platforms (2/34, 6%), and tablets (2/34, 6%).

**Table 3:** Summary of studies on digital personalization for hypertension (n=34).

First author	Study design	Sample size	Digital technology	Primary outcomes
Beran et al [20]	Mixed methods	450	Mobile phone and BP^a^ monitor	BP
Blood et al [21]	Cohort	10,803	Mobile phone and BP monitor	BP
Bosworth et al [22]	RCT^b^	428	Mobile phone and BP monitor	CVD^c^ risk score
Brewer et al [23]	Mixed methods	16	Mobile phone	BP
Chandler et al [24]	RCT	56	Mobile phone, BP monitor, and electronic medication trays and pill boxes	BP
Choudhry et al [25]	RCT	4078	Mobile phone	Medication adherence
David et al [26]	Secondary analysis of RCT	231	Mobile phone and BP monitor	BP
Davidson et al [27]	RCT	38	Mobile phone, BP monitor, electronic medication tray	BP
Glynn et al [28]	Qualitative	50	Views on technology	Views on technology
Guthrie et al [29]	Cohort	172	Mobile phone and BP monitor	BP
Hellem et al [30]	Qualitative	86	Mobile phone	Feedback on the design of the mobile app intervention
Jeong et al [31]	Pilot RCT	35	Mobile phone and BP monitor	Health behavior and BP
Kario et al [32]	RCT	390	Mobile phone and BP monitor	BP
Kassavou et al [33]	RCT	135	Mobile phone	Medication adherence
Klein et al [34]	Cohort	38	Mobile phone	Medication adherence
Leitner et al [35]	RCT	38	Wearable device, mobile phone, and BP monitor	BP
Lewinski et al [36]	Pilot RCT	118	Mobile phone	BP
Lv et al [37]	Pre-post	149	Mobile phone, BP monitor web-based system, and wearable device	BP
McBride et al [38]	Qualitative	11	Mobile phone and BP monitor	Feedback using app
McGillicuddy et al [39]	Pilot RCT	20	Mobile phone, BP monitor, electronic medication tray	BP and medication adherence
Naqvi et al [40]	Pilot RCT	50	Electronic tablet and BP monitor	BP
Payne Riches et al [41]	Pilot RCT	47	Mobile phone	Follow-up, fidelity, and app use
Petrella et al [42]	RCT	149	Mobile phone, BP monitor, and wearable device	BP
Rodriguez et al [43]	RCT	544	Mobile phone	BP
Rodriguez et al [44]	RCT	533	Mobile phone	Stage of change and DASH^d^ score
Saleh et al [45]	Mixed methods	606	Mobile phone	Patient satisfaction
Schoenthaler et al [46]	Pilot RCT	42	Electronic tablet	Intervention acceptance and BP
Shea et al [47]	Pilot RCT	140	Mobile phone	BP
Steinberg et al [48]	Pilot RCT	140	Mobile phone	Diet tracking and DASH score
Thiboutot et al [49]	RCT	500	Website	BP
Van Emmenis et al [50]	Qualitative	20	Mobile phone	Views on the mobile app intervention
Wang et al [51]	Pilot RCT	49	Mobile phone and BP monitor	Feasibility of app use
Willis et al [52]	Cohort	7752	Mobile phone and BP monitor	BP
Zhang et al [53]	RCT	192	Wearable device and mobile phone	BP

^a^BP: blood pressure.

^b^RCT: randomized controlled trial.

^c^CVD: cardiovascular disease.

^d^DASH: dietary approaches to stop hypertension.

Of the 34 studies, 4 (12%) qualitative studies highlighted participants’ perceived importance of personalization in digital interventions. Participants emphasized the need for digital interventions to be customizable, allowing for tailoring based on their preferences, such as personalizing functions for SMS text messages, the inclusion of all medications in the app, and the ability to adjust reminder settings [28,50]. A study on the design of a mobile app intervention found distinct differences in preferences between participants from a federally qualified health center and a university cardiovascular clinic; the former group placed greater value on social support themes and addressing health-related social needs [30]. McBride et al [38] found that participants using a mobile app for home BP monitoring felt the app’s visual feedback and medication reminders improved their understanding of their condition as well as their sense of control and responsibility, resulting in improved self-management practices.

#### Personalization Characteristics of the Digital Interventions

##### Modalities and Approaches

For the 30 studies that tested a personalized digital intervention (Table 4), the modality of intervention delivery occurred exclusively through mobile apps for half of the studies (n=16, 53%), phone calls only (n=5, 17%), SMS text messages only (n=2, 7%), and web- or tablet-based only (n=2, 7%). Of the 30 studies, 6 (20%) used ≥2 modalities (ie, phone calls, texts, emails, and mobile apps) for intervention delivery. Of the 30 studies, 19 (63%) used personnel (eg, case managers, patient navigators, and clinicians) for some aspect of the intervention design and delivery, while 11 (37%) studies relied solely on digital technology for intervention delivery.

**Table 4:** Personalization characteristics of the digital interventions (n=30).

First author	Personnel	Human vs automated tailoring	Personalized elements of intervention	Study findings
Beran et al [20]	Pharmacists	Hybrid	Tailored education Individualized medication management	CS^a^
Blood et al [21]	Pharmacists, patient navigators, and NPs^b^ and MDs^c^	Hybrid	Tailored education Individualized medication management	SS^d^
Bosworth et al [22]	Pharmacists	Human	Tailored education Individualized medication management	NS^e^
Brewer et al [23]	Community health workers and clinicians	Hybrid	BP^f^ and medication tracking on an app Culturally tailored education Assessment of SDoH^g^ needs and referrals for services (housing and utilities)	CS
Chandler et al [24]	—^h^	Automated	Reminders, feedback, and motivational messages based on treatment adherence, and values, beliefs, or goals	SS
Choudhry et al [25]	Pharmacists	Hybrid	Tailored education Assessment of SDoH needs and referrals for social work and affordable prescription options	SS
David et al [26]	—	Automated	Tailored education Personalized feedback based on BP, goals, and treatment adherence	SS
Davidson et al [27]	—	Automated	Culturally tailored SMS text messages based on treatment adherence and values, beliefs, or goals	SS
Guthrie et al [29]	Multidisciplinary team	Hybrid	AI^i^-assisted tailored feedback and education on self-management and treatment adherence	SS
Jeong et al [31]	Nurses	Human	Tailored education addressing self-management behaviors and treatment adherence	CS
Kario et al [32]	Health professionals and chat-bot based virtual nurses	Hybrid	Personalized lifestyle program based on age, sex, lifestyle, social background, and behavior patterns	SS
Kassavou et al [33]	Clinicians	Automated	Tailored messages on improving medication adherence based on patient beliefs, attitudes, self-efficacy, and emotional state Medication refill reminders sent via SMS text messages	SS
Klein et al [34]	—	Automated	Tailored SMS text messages to improve medication adherence comprising educational and motivational content	CS
Leitner et al [35]	—	Automated	AI-driven personalized lifestyle recommendations based on clinical, behavior, and psychological data	CS
Lewinski et al [36]	Case manager	Human	Tailored education on self-management behaviors and treatment adherence	NS
Lv et al [37]	Case manager, dietitian, and pharmacist	Human	Individualized BP management plan Tailored feedback on BP, medicines, weight, steps, and diet	SS
McGillicuddy et al [39]	—	Automated	Tailored reminders, feedback, and summary reports including positive reinforcement and suggestions for improvement Tailored to medication dosing schedule and BP goals	SS
Naqvi et al [40]	NPs or MDs and pharmacists	Human	Individualized consultations regarding symptoms and medication changes BP infographics tailored to BP at discharge, available in English and Spanish	SS
Payne Riches et al [41]	Clinicians	Hybrid	Individualized goal setting and feedback on participants’ food choices to help them identify lower-salt options	CS
Petrella et al [42]	Exercise specialists	Human	Tailored exercise program prescribed by an exercise specialist based on fitness level	NS
Rodriguez et al [43]	Counselors	Human	Tailored counseling based on the stage of change for adherence to exercise, diet, and medication adherence Individualized assessment of barriers to behavior changes and solutions	SS
Rodriguez et al [44]	Counselors	Human	Tailored counseling based on the stage of change for DASH^j^ diet, exercise, and medication adherence	SS
Saleh et al [45]	—	Automated	Personalized reminders for appointments, laboratory tests, and examinations Content tailored to language and health literacy	SS
Schoenthaler et al [46]	Research assistant	Automated	Personalized list of adherence intervention strategies based on unique barriers to treatment adherence	SS
Shea et al [47]	—	Automated	Tailored educational texts based on participants’ priority topics and most recent BP reading	CS
Steinberg et al [48]	—	Automated	Tailored messages based on adherence to the DASH diet Content was tailored for women and provided behavioral tips to reinforce dietary change and provide social support	NS
Thiboutot et al [49]	—	Automated	Customized feedback and recommendations on questions to discuss with their health care providers	NS
Wang et al [51]	—	Automated	Individual monitoring of treatment response and tailored recommendations for follow-up based on BP levels	CS
Willis et al [52]	MDs	Human	Individualized remote treatment based on BP levels	SS
Zhang et al [53]	Health facilitators	Human	Tailored feedback on self-management behaviors	CS

^a^CS: clinically significant but not statistically significant.

^b^NP: nurse practitioner.

^c^MD: medical doctor.

^d^SS: statistically significant.

^e^NS: no statistical or clinical significance.

^f^BP: blood pressure.

^g^SDoH: social determinants of health.

^h^Not applicable.

^i^AI: artificial intelligence.

^j^DASH: dietary approaches to stop hypertension.

Personalization strategies varied, with 13 (43%) of the 30 studies using a fully automated personalization approach without the need for human involvement. For example, interventions used automated SMS text messages to improve treatment adherence and provide feedback to participants [24,26,34] or used an artificial intelligence–driven system to generate weekly tailored lifestyle recommendations [35]. Of the 30 studies, 10 (33%) used human-driven personalization, in which study personnel were the primary avenues for personalization of the intervention. For example, nurses and case managers offered patients personalized education on self-management behaviors and treatment adherence [31,36], and pharmacists and physicians provided individualized medication adjustments based on treatment response [22,52]. Of the 30 studies, 7 (23%) used a hybrid approach (ie, both the technology and human study personnel contributed to the personalization of the intervention).

Most personalized intervention studies (25/30, 83%) had either statistically significant positive outcomes (16/30, 53%) or improved or clinically meaningful outcomes, although not statistically significant (9/30, 30%). The studies with nonsignificant, yet positive findings tended to be pilot studies or had small sample sizes (ie, <40 participants). Among the 25 studies with significant positive outcomes, 16 (64%) involved personnel in intervention delivery, with involvement levels varying from comprehensive multidisciplinary teams (pharmacists, nurse practitioners, physicians, and patient navigators) offering patient education, medication management, and monitoring [21], to minimal, such as research assistants giving participants tablet use instructions [46]. In addition, 48% (12/25) of these studies used an automated tailoring approach, followed by 28% (7/25) of the studies using human-led tailoring and 24% (6/25) using a combination of both human-led and automated tailoring.

##### Personalized Elements of the Interventions

Personalized interventions included tailored education on self-management (ie, medication adherence, diet, and exercise), along with motivational messages and feedback aligned with each participant’s adherence, values, beliefs, and goals. For instance, a culturally adapted mobile health intervention sent the following message to a Hispanic man hoping to find a wife and start a family: “Get back on track taking your meds for building that stronger, healthier body for when you meet that special woman” [24]. Studies also provided personalized reminders for medications, appointments, laboratory tests, and individualized medication management based on treatment adherence and BP levels. Of the 30 studies, only 2 (7%) provided assessment and referrals for social determinants of health needs (ie, housing, utilities, and affordable prescription options) [23,25].

##### Data Used for Personalization

Tailored intervention studies used a range of data types for personalization of the intervention (Table 5). From 8 categories of data available for personalization (eg, demographic, clinical, and behavioral), just over half the studies (16/30, 53%) used ≥3 types of data, with 14 (47%) studies using ≤2 data types. Of the 30 studies, 6 (20%) used 5 categories of data for personalization, with no studies using >5 data types. The 3 most common types of data used for personalization were behavioral (28/30, 93%), clinical (24/30, 80%), and psychological (17/30, 57%). Behavioral data were typically used to monitor patients’ adherence to key behaviors relevant to HTN management, such as taking medication, physical activity, and diet. Collecting real-time information on participants’ health behaviors allowed for individualized feedback in many studies. For example, in the study by Steinberg et al [48], participants reported their food intake and could receive texts with feedback and guidance, such as “You did best with reducing saturated fat and boosting your fiber intake and seemed to struggle with getting enough potassium and magnesium...to get more magnesium, try dried fruit as a snack!” Similarly, clinical data such as BP or medication-related information were used to offer feedback tailored to participant’s clinical metrics. For instance, Wang et al [51] developed a telehealth system that used decision rules to adjust care based on BP levels; if BP was optimal and participants were adherent to medications without side effects, follow-up appointments could be deferred and medication refills could be automatically prescribed. Psychological data included information on participants’ attitudes, beliefs, values, and goals. For example, a few studies assessed participants’ “stage of change” [43,44] and readiness to modify behaviors [25], while others took into account their values, attitudes, and beliefs [24,27,34] or their perceived barriers to self-management [25,36,43,46].

**Table 5:** Data types used for personalization in digital interventions (n=30).

Study	Demographic (n=4)	Socioeconomic (n=6)	Clinical (n=24)	Behavioral (n=28)	Psychologic (n=17)	Health literacy (n=6)	Physical environment	Cultural (n=4)
Beran et al [20]			✓	✓				
Blood et al [21]			✓					
Bosworth et al [22]	✓		✓	✓	✓	✓		
Brewer et al [23]		✓	✓	✓		✓		✓
Chandler et al [24]			✓	✓	✓			✓
Choudhry et al [25]		✓	✓	✓	✓			
David et al [26]			✓	✓				
Davidson et al [27]			✓	✓	✓	✓		✓
Guthrie et al [29]			✓	✓	✓			
Jeong et al [31]		✓	✓	✓	✓			
Kario et al [32]	✓	✓	✓	✓	✓			
Kassavou et al [33]				✓	✓			
Klein et al [34]				✓	✓			
Leitner et al [35]			✓	✓	✓			
Lewinski et al [36]		✓	✓	✓	✓	✓		
Lv et al [37]			✓	✓	✓			
McGillicuddy et al [39]			✓	✓	✓			
Naqvi et al [40]	✓		✓	✓				
Payne Riches et al [41]				✓	✓			
Petrella et al [42]			✓	✓				
Rodriguez et al [43]			✓	✓	✓			
Rodriguez et al [44]				✓	✓			
Saleh et al [45]			✓	✓				
Schoenthaler et al [46]		✓		✓	✓			✓
Shea et al [47]			✓	✓				
Steinberg et al [48]	✓			✓				
Thiboutot et al [49]			✓					
Wang et al [51]			✓	✓				
Willis et al [52]			✓	✓				
Zhang et al [53]			✓	✓				

The other types of data used for personalization were all used by less than one-third of the studies: health literacy (6/30, 20%), social and economic (6/30, 20%), demographics (4/30, 13%), and cultural (4/30, 13%). No studies obtained physical environment data for personalization of the intervention. For health literacy, the prevailing strategy involved designing interventions for a low-literacy population as a whole, rather than tailoring it at the individual participant level, and none of the studies tailored interventions to the level of digital literacy [36,45,54]. Similarly, studies implementing cultural tailoring primarily focused on specific populations as opposed to tailoring at the individual level. For example, Brewer et al [23] and Schoenthaler et al [46] both conducted studies with only Black participants and offered culturally tailored content geared for this population, such as education on the impact of racism and discrimination on BP. However, not all studies applied cultural tailoring broadly. Davidson et al [27] assessed cultural values and beliefs at an individual level, sending customized SMS text messages based on these insights.

### Digital Phenotyping

There were 4 studies with a precision health focus on phenotyping [19,55-57] using a range of demographic, behavioral, clinical, and genetic data to apply phenotyping in diverse ways to enhance understanding and management of HTN (Table 6). Sample sizes ranged from 13 for a qualitative study [57] to 764,135 in a whole-genome sequencing analysis [19]. Digital tools used included mobile phones (2/4, 50%), web platform (1/4, 25%), electronic health record (EHR; 1/4, 25%), machine learning (ML) algorithms (1/4, 25%), BP monitor (1/4, 25%), and genomic databases (1/4, 25%).

**Table 6:** Summary of the studies on digital phenotyping for HTN^a^.

First author	Study design	Sample size	Digital technology	Description of the study
Bakre et al [55]	Secondary data analysis	11,934	Mobile phone and web platform	The study analyzed demographic, dietary, and clinical data of participants with stage 2 HTN who used a digital nutrition platform to identify characteristics associated with greater reductions in BP^b^.
Chen et al [56]	Secondary data analysis	2521	EHR^c^ and ML^d^ algorithm	The study developed an ML framework to identify predictive features and cluster patients with HTN into 4 clinically meaningful groups of disease severity.
Hellem et al [57]	Qualitative	13	Mobile phone and BP monitor	The study examined participants’ determinants of engagement from a digital intervention through interviews, phenotyping participants into high engagers, low engagers, and early enders.
Kelly et al [19]	Genome sequencing	764,135	Genomic databases	This is the first study to identify a promising but unconfirmed intergenic rare variant associated with BP. This variant lowered SBP^e^ by an average of 33 mm Hg in carriers compared with noncarriers, and all participants were of Asian ancestry.

^a^HTN: hypertension.

^b^BP: blood pressure.

^c^EHR: electronic health record.

^d^ML: machine learning.

^e^SBP: systolic blood pressure.

Of the 4 studies, 2 (50%) used secondary data analysis to apply phenotyping. Bakre et al [55] applied phenotyping by analyzing demographic, dietary, and clinical data of participants with stage 2 HTN who used a digital nutrition platform, identifying characteristics associated with a decrease in BP. They found that participants who achieved greater BP reductions had higher reductions in weight and dietary improvements, suggesting the effectiveness of digital nutrition guidance on management of HTN [55]. Chen et al [56] used EHR data to develop an ML framework to phenotype patients with HTN into 4 clinically meaningful groups, aiming to improve personalization of care and resource allocation. They used demographic and clinical data for cluster analysis, identifying 2 groups with mild disease and 2 with more severe disease profiles.

The qualitative study by Hellem et al [57] explored participants’ determinants of engagement from a SMS text messaging intervention to reduce BP among patients with HTN. Through interviews with participants purposely sampled from 3 engagement categories—high engagers, low engagers, and early enders—this study phenotyped individuals based on a mix of social, psychological, and environmental factors, including digital literacy and access. They found that high engagers had a better understanding of the intervention, the least number of social needs, and the greatest social support. In contrast, early enders and low engagers expressed greater amounts of social needs and less social support [57]. Finally, the study by Kelly et al [19] phenotyped participants at a genetic level, offering a direct link between genotype and phenotypic expression of BP, presenting a road map for targeted genetic interventions for personalized management of HTN.

### Digital Prediction Tools

There were 8 studies with a precision health focus on prediction models (Table 7). These studies collectively explored advanced ML techniques for personalized risk prediction for HTN management. Study designs were primarily ML model development (7/8, 88%), with 1 (13%) proof-of-concept study [58] and 1 (13%) hybrid design comprising both ML model development and RCT [59]. Sample sizes varied from 7 for the proof-of-concept study [58] to 245,499 for the study using EHR data [60]. Digital technologies used for these studies included the following: ML algorithms (8/8, 100%), EHR (5/8, 63%), wearable devices (4/8, 50%), mobile phone and web platform (1/8, 13%), and BP monitor (1/8, 13%). Various ML models were used for each of these studies, with half of the studies using random forest models (4/8, 50%), with others including online recurrent extreme learning machine and long short-term memory.

**Table 7:** Summary of the studies on prediction models for HTN^a^ (n=8).

First author	Study design	Sample size	Digital technology	Study description	ML^b^ models
Abrar et al [61]	ML model development	35	ML algorithms	Development of a personalized BP^c^ prediction model tailored to individual physiology and lifestyle factors	OR-ELM^d^
Bernal et al [58]	Proof of concept	7	ML algorithms, wearable device, mobile phone, and web platform	Development of an intelligent web-based ecosystem that integrates clinical, behavioral, and environmental data to predict adverse high BP events	Bayesian ridge, support vector regression, and random forest
Bertsimas et al [62]	ML model development	19,926	ML algorithms and EHR^e^	Development of ensemble ML models for personalized predictions and treatment recommendations, with significant improvements in HTN through optimal treatment prescriptions based on individual patient data	Classification models (eg, multivariate logistic regression and random forests) and regression models (eg, support vector regression and optimal regression trees)
Cano et al [63]	ML model development	86	ML algorithms, wearable device, and EHR	Development of a system for improved discrimination between healthy individuals, individuals with prehypertension, and individuals with HTN using PPG^f^ and ECG^g^ signals, focusing on improving the accuracy of HTN detection	A total of 37 different classification models were used: logistic regression, support vector machines, and nearest neighbors, with the Coarse Tree model achieving the highest discrimination
Chiang et al [59]	RCT^h^ and ML model development	25	ML algorithms, wearable device, and BP monitor	Development of a personalized BP model for each individual using clinical and behavioral data, identifying the most important lifestyle factors impacting BP trend, and providing precise recommendations for HTN management	Random forest with Shapley value–based feature selection model outperformed other models in prediction accuracy
Hu et al [64]	ML model development	42,792	ML algorithms, wearable device, and EHR	Development of an ML model for personalized antihypertensive medication classes for patients with HTN using a robust algorithm that accommodates outliers in EHR data	The distributionally linear regression–informed k-nearest neighbors model resulted in a 14.22 mm Hg reduction in SBP^i^ on average
Jimeng et al [65]	ML model development	1294	ML algorithms and EHR	Development of ML model to predict transition points in HTN control status, aiming to inform personalized management strategies	Logistic regression, naïve Bayes, and random forests
Ye et al [60]	ML model development	245,499	ML algorithms and EHR	Development of deep learning models to predict individualized HTN treatment pathways based on EHR data	LSTM^j^ and bidirectional LSTM

^a^HTN: hypertension.

^b^ML: machine learning.

^c^BP: blood pressure.

^d^OR-ELM: online recurrent extreme learning machine.

^e^EHR: electronic health record.

^f^PPG: photoplethysmography.

^g^ECG: electrocardiogram.

^h^RCT: randomized controlled trial.

^i^SBP: systolic blood pressure.

^j^LTSM: long short-term memory.

Emphasizing personalized health care, these studies demonstrate improved accuracy in HTN control by tailoring recommendations to individual patient profiles, challenging traditional one-size-fits-all approaches. One key difference lies in the scale and nature of the datasets used, with some researchers analyzing extensive patient data over extended periods [62], whereas others focus on smaller, more controlled patient groups [59]. In addition, the scope of each study varies; some studies aim to improve BP prediction accuracy, while others focus on optimizing treatment pathways [64], integrating lifestyle data [59], or providing real-time [58] and personalized health care recommendations [59].

## Discussion

### Principal Findings

This scoping review provided a comprehensive mapping of the literature on the trends and gaps in digital precision health research for the management of HTN in adults. Analysis of 46 studies identified 3 main categories of precision health focus: personalization, phenotyping, and prediction. The predominance of personalization (34/46, 74% of studies), compared to prediction (8/46, 17%) and phenotyping (4/46, 9%), highlights a strong emphasis on tailored interventions but reveals limited attention to predictive modeling and comprehensive phenotyping, which are essential for predicting HTN outcomes and identifying and categorizing patient subgroups.

### Gaps in Digital Precision Health Research

The review revealed geographic, disciplinary, and demographic gaps in digital precision health research for HTN. Most studies (30/46, 65%) reviewed were conducted in the United States, followed by Europe (7/46, 15%), Asia (6/46, 13%), and South America (1/46, 2%), with no studies from Africa or Oceania. This contrasts with high HTN prevalence rates in Africa and Oceania, underscoring the need for more region-specific research to better understand local challenges and develop culturally sensitive and effective strategies for HTN prevention and management [5].

Disciplinary gaps were evident, with >60% (29/46) of the studies originating solely from the medical and health sciences. Less than a third of the studies (15/46) involved interdisciplinary teams, highlighting the need for collaborative efforts across disciplines such as engineering, informatics, and social sciences. Transdisciplinary research, in which team members from diverse fields develop unified conceptual frameworks that transcend their respective disciplinary views, is essential for advancing precision health research in HTN [66].

Demographic reporting was inconsistent and insufficient across studies. While 96% (44/46) of the studies reported participants’ gender and age, 45% (21/46) did not report race and ethnicity, or only did so partially. Of those that reported race and ethnicity, the median percentages of participant representation were skewed heavily toward non-Hispanic White (51%), followed by African American (40%), with underrepresentation of Hispanic or Latinx (5%) and Asian (1%) groups. This lack of diversity raises concerns about generalizability of findings, concern for bias, and underscores the need to for transparency in reporting demographic data, which is especially salient in the context of precision health [67].

### Personalized Digital Interventions: Tools, Modalities, and Data Types

Mobile phones were the predominant tools for digital HTN interventions (30/34, 88%), while only 50% (17/34) incorporated home BP monitors and 12% (4/34) used wearable devices. This highlights an opportunity to integrate wearable sensor devices for richer data collection (eg, sleep, heart rate, and physical activity), fostering a more holistic understanding of HTN and its management. Wearable BP monitoring devices is a growing area of research and offers noninvasive, continuous BP monitoring through methods such as photoplethysmography [68]. These devices offer a more comprehensive view of cardiovascular health over time, enabling continuous monitoring, personalized treatment plans, and enhanced predictive capabilities for early intervention. However, further research is needed to enhance their accuracy, calibration, and validation for clinical use [68].

Traditional communication methods such as phone calls and texting were still used (5/30, 17% and 2/30, 7% of studies, respectively), demonstrating their relevance especially for reaching underserved populations with limited digital literacy or access to advanced technology. However, only 20% (6/30) of the studies used multiple intervention delivery modalities (ie, phone calls, texting, and apps), suggesting the need for more integrative approaches to improve access, engagement, and efficacy of digital interventions by catering to different user preferences and needs.

Most studies used behavioral (28/30, 93%), clinical (24/30, 80%), and psychological (17/30, 57%) data for personalization, but only 7% (2/30) of the studies incorporated social determinants such as housing needs or prescription affordability [23,25]. No studies included physical environment or digital literacy data, highlighting key gaps in personalized digital interventions for HTN. Prior studies have documented poor digital literacy and lack of support and training as significant barriers to the use of digital health tools by populations considered disadvantaged [69]. This underscores the need to incorporate diverse sources of data to provide the additional context that can help address disparities in digital intervention engagement and efficacy. Health equity–focused frameworks, such as the Digital Health Equity framework [70], can be used to guide the development and implementation of digital interventions to ensure holistic data collection and personalization, mitigate intervention-generated inequalities, and better address existing health disparities in HTN.

### Defining Personalization Strategies

This review identified a critical need for greater clarity and specificity in the use of terminology such as “personalization” or “tailoring” to improve the understanding and application of tailored interventions. As personalization is so broadly defined, it remains challenging to assess the most effective characteristics of personalization for digital interventions. For example, Steinberg et al [48] wrote that “only 28% of participants said they felt the texts were personalized, despite the use of an algorithm designed to personalize messages about intake of specific DASH nutrients.” Such findings indicate the importance of research that specifically delineates which strategies and approaches to personalization are most impactful for users; specifically, there is a need for tools to characterize these approaches to personalization. Fan and Poole [71] provide a useful classification scheme for how personalization can be implemented. Two of the dimensions they describe, in particular, are highly relevant to our corpus: the target of the intervention (either individual or categorical) and the automation approach (either explicit or configured by the user or implicit or automated by the system).

Studies in this review used both individual and categorical targets. For instance, in the study by Kario et al [32], personalization was implemented broadly to group characteristics through tailored BP infographics developed for the target population. This is in contrast to personalization at the individual level, such as in the study by Brewer et al [23], in which participants were assessed for individual social needs and referred for needed services (ie, housing and utilities). While categorical-level personalization strategies can be effective and impactful, we caution against their widespread use in personalization discussions, as precision health primarily focuses on individual-level differences within a patient population. Further research is needed to better understand the characteristics and levels of personalization that are most effective for diverse populations.

In addition, the distinction of whether personalization is automated or human driven is an important lens in understanding an intervention and its effects, and studies in this review leveraged both these approaches as well as hybrid versions of them. While automated models can streamline interventions, they may lack the adaptability of human-led approaches.

Hybrid methods, combining automated and human input, could address the limitations of each approach alone. For example, Schoenthaler et al [46] mentioned the following in their paper: “despite high acceptability, one-third of intervention participants recommended including a health educator as an adjunct to the mHealth intervention, suggesting that some in-person contact is important and could not be replaced by the design of this intervention.” Several papers in our review demonstrate hybrid approaches to personalization, which could guide future research. For example, the intervention developed by Choudhry et al [25] involved a pharmacist-led, semistructured consultation with specific guidelines for mapping barriers to adherence and developing corresponding response strategies. This human-driven, semistructured consultation is paired with automated, system-driven intervention elements, such as SMS text messages. Such hybrid approaches to the design of digital health interventions present a promising avenue for future research.

### Digital Phenotyping

The 4 studies focusing on phenotyping within digital precision HTN management illuminate the potential for leveraging a wide spectrum of data—demographic, clinical, behavioral, and genetic—to identify unique phenotypes of participants with HTN to tailor treatment strategies. Multiple study designs, including qualitative interviews and whole-genome analysis, have been used to enhance understanding of HTN. Leveraging secondary data from EHRs and past RCTs for analysis using ML algorithms can help cluster phenotypes of patients with HTN and explain variations in treatment response [72]. Classifying individuals into meaningful groups based on HTN phenotypes is a crucial step toward personalized care and advancing precision health.

By contrast, qualitative approaches, despite having smaller sample sizes, have the ability to provide a more contextual, holistic, and nuanced understanding of the social and psychological determinants of HTN treatment adherence and outcomes from digital interventions, which is crucial for understanding disparities. Future areas of research should integrate the use of multiple sources of data, such as surveys, wearable devices, remote BP monitoring, and ecological momentary assessments, to derive digital phenotypes of participants’ self-management behaviors and provide more effective interventions [73].

### Prediction Models

The 8 studies with a precision health focus on prediction models for digital HTN management highlight significant advancements and challenges in the field. These studies collectively demonstrate the innovative use of advanced ML techniques for personalized risk prediction and intervention strategies. Using a wide array of digital tools, including EHR data, wearable devices, mobile platforms, and remote monitoring, these studies are shifting the paradigm beyond one-size-fits-all approaches to care to offer more personalized and preventive care. ML models can help identify factors to predict individuals’ risk of HTN, potentially preventing the development of high BP through early intervention. There are a growing number of studies using diverse ML methods (eg, support vector machine, deep learning, and XGBoost) and data types (eg, genetic, behavioral, and sociodemographic) to accurately predict the development of HTN [74-76].

Despite the potential of prediction models to transform HTN care, these studies also illuminate the hurdles that must be overcome to realize this potential fully. For instance, the generalizability of findings is a significant concern, with varying sample sizes and potential biases from using specific datasets that may not be representative of the population at large. A recent analysis of 63 HTN research studies using ML methods found that only 46% of studies described the participant demographics, and none of the studies provided a rigorous assessment of algorithmic bias, with only 6 studies acknowledging a risk of bias [77]. Algorithmic bias occurs when the diversity of the input dataset used for model development does not match that of the target population, resulting in inaccurate predictions for underrepresented groups and disparities in outcomes [77]. The effectiveness of ML models to accurately predict risk and personalize care relies heavily on the quality of data on which they are trained. The lack of representativeness of participants in training data for these models poses significant concerns regarding their generalizability and potential for bias [67]. To address these limitations, reporting guidelines such as CONSORT-AI (Consolidated Standards of Reporting Trials–Artificial Intelligence) or TRIPOD-AI (Transparent Reporting of a Multivariable Prediction Model for Individual Prognosis or Diagnosis–Artificial Intelligence) should be used when reporting ML prediction models for HTN; these guidelines provide specific criteria for authors to follow, ensuring transparency, completeness, and standardization in study reporting [78,79]. These guidelines emphasize detailed reporting of model development, training, validation, performance metrics, data sources, and patient characteristics which enable comparisons across studies. Furthermore, the prediction models reviewed focused primarily on development, underscoring a key research gap in model deployment and real-world implementation. Advancing precision health in HTN management requires research on best practices for deploying, evaluating, and monitoring models in clinical care.

### Strengths and Limitations

This scoping review on digital precision HTN management followed a systematic approach based on the framework developed by Arksey and O’Malley [17], ensuring a rigorous and comprehensive exploration of the field. By adopting a precision health lens, the review covers a broad spectrum of studies, including personalized interventions, prediction models, and phenotyping, offering valuable insights into these areas. The detailed analysis of personalized intervention studies, particularly regarding the characteristics of personalization and data used for personalization, adds depth to the understanding of how digital tools can optimize HTN care. In addition, the interdisciplinary composition of the review team, drawing expertise from nursing, informatics, and computer science, further strengthens this review by incorporating diverse perspectives.

However, the broad scope of this review and the heterogeneity of included studies introduce certain limitations. For example, a detailed examination of intervention effectiveness and statistical significance was limited due to diverse study designs, such as underpowered pilot studies or qualitative designs. The absence of a quality assessment for the included studies may also compromise the validity of the conclusions drawn, as it overlooks methodological rigor and potential biases within studies. Finally, our search terms included “precision health” and only the “personalization” derivatives (eg, *precision medicine* OR *personalized* OR *tailored* OR *individualized*), which could have limited the number of studies on prediction models or phenotyping. However, the review team chose to include the studies on prediction models and phenotyping as these were considered to be important aspects of precision health research. Future reviews could emphasize a more comprehensive search of these specific aspects of precision health research for digital HTN management.

### Conclusions

This scoping review of 46 studies synthesized the current state of precision health research encompassing personalization, phenotyping, and prediction using digital health tools for the management of HTN in adults. The findings from our review demonstrate that the majority (34/46, 74%) of the included studies had a precision health focus of personalization in digital HTN management, followed by prediction models and phenotyping. Our analysis highlighted significant gaps in reporting of participant race and ethnicity data, geographic distribution of research in digital HTN management, and use of wearable devices for capturing passive data. Furthermore, the analysis of personalization characteristics in interventions underscores the need for the integration of multiple sources of data for personalization, such as social determinants of health, physical environment, and digital literacy, to promote health equity. There is a need for more precision in the use of the term “personalization” as well as further research that explores the impact of specific types of personalization on health outcomes. Finally, greater interdisciplinary collaboration and ultimately a transdisciplinary approach are needed to meaningfully advance the field of precision health for HTN risk prediction, prevention, and management.

## Appendix

Multimedia Appendix 1 PRISMA (Preferred Reporting Items for Systematic Reviews and Meta-Analyses) checklist.

Multimedia Appendix 2 Database search terms.

Multimedia Appendix 3 Characteristics of the included studies.

## Abbreviations

BP: blood pressure
CONSORT-AI: Consolidated Standards of Reporting Trials–Artificial Intelligence
EHR: electronic health record
HTN: hypertension
ML: machine learning
PRISMA-ScR: Preferred Reporting Items for Systematic Reviews and Meta-Analyses
RCT: randomized controlled trial
TRIPOD-AI: Transparent Reporting of a Multivariable Prediction Model for Individual Prognosis or Diagnosis–Artificial Intelligence
