# Supplementary material for: Trends and Gaps in Digital Precision Hypertension Management: Scoping Review
Source: J Med Internet Res. 2025 Feb 10;27:e59841. doi: 10.2196/59841 (PMC11851032; doi:10.2196/59841)
Supplement: Multimedia Appendix 2 [file jmir_v27i1e59841_app2.docx]

Multimedia Appendix 2. Database Search Terms

| Database | Search Terms |
| --- | --- |
| PubMed | ((("precision medicine"[MeSH Terms] OR "precision medicine"[Title/Abstract] OR "precision health"[Title/Abstract] OR "tailored"[Title/Abstract] OR "individualized"[Title/Abstract] OR "personalized"[Title/Abstract])) AND (("telemedicine"[MeSH Terms] OR "telemedicine"[Title/Abstract] OR "virtual medicine"[Title/Abstract] OR "telehealth"[Title/Abstract] OR "mobile health"[Title/Abstract] OR "mHealth"[Title/Abstract] OR "eHealth"[Title/Abstract] OR "electronic health"[Title/Abstract] OR "digital health"[Title/Abstract] OR "digital interventions"[Title/Abstract:~5] OR "mHealth interventions"[Title/Abstract:~5] OR "electronic interventions"[Title/Abstract:~5] OR "remote healthcare delivery"[Title/Abstract] OR "mobile applications"[MeSH Terms] OR "mobile applications"[Title/Abstract] ) OR smartphone[MeSH Terms] OR (smartphone[Title/Abstract] OR "wearable electronic devices"[MeSH Terms] OR "wearable electronic device*"[Title/Abstract]))) AND ((hypertension[MeSH Terms]) OR hypertension [tiab] OR (high blood pressure[Title/Abstract])) |
| CINAHL | MH ( "precision medicine" OR "precision health" OR personalized or personalised or tailored or individualized ) OR TI ( "precision medicine" OR "precision health" OR personalized or personalised or tailored or individualized ) OR AB ( "precision medicine" OR "precision health" OR personalized or personalised or tailored or individualized ) AND MH ( telemedicine OR telehealth OR virtual medicine OR “mobile health” OR mHealth OR eHealth OR “digital health” OR “electornic health” OR “digital interventions” OR “mHealth interventions” OR “mobile applications” OR “smartphone” OR wearable* OR "wearable electronic device*) OR TI ( telemedicine OR telehealth OR virtual medicine OR “mobile health” OR mHealth OR eHealth OR “digital health” OR “electronic health” OR “digital interventions” OR “mHealth interventions” OR “mobile applications” OR “smartphone” OR wearable* OR "wearable electronic device*) OR AB ( telemedicine OR telehealth OR virtual medicine OR “mobile health” OR mHealth OR eHealth OR “digital health” OR “electronic health” OR “digital interventions” OR “mHealth interventions” OR “mobile applications” OR “smartphone” OR wearable* OR "wearable electronic device*)) AND MH ( hypertension or high blood pressure or elevated blood pressure or htn or hypertensive ) OR TI ( hypertension or high blood pressure or elevated blood pressure or htn or hypertensive ) OR AB ( hypertension or high blood pressure or elevated blood pressure or htn or hypertensive ) |
| Web of Science | TS=("precision medicine" OR "precision health" OR tailored OR individualized OR personalized) AND TS=(telemedicine OR telehealth OR "virtual medicine" OR "virtual health" OR "mobile health" OR mHealth OR eHealth OR "electronic health" OR "digital health" OR "digital interventions" OR "mHealth interventions" OR "electronic interventions" OR "remote healthcare" OR "mobile applications" OR smartphone OR wearable* OR wearable electronic device*) AND TS=(hypertension OR "high blood pressure" OR "elevated blood pressure" OR hypertensive OR HTN) |
| Inspec | "precision medicine" OR "precision health" OR personalized or personalised or tailored or individualized AND telemedicine OR telehealth OR "virtual medicine" OR "virtual health" OR "mobile health" OR mHealth OR eHealth OR "electronic health" OR "digital health" OR "digital interventions" OR "mHealth interventions" OR "electronic interventions" OR "remote healthcare" OR "mobile applications" OR smartphone OR wearables OR wearable electronic devices AND hypertension OR "high blood pressure" OR "elevated blood pressure" OR hypertensive |
| Embase | 'precision medicine'/exp OR 'precision medicine' OR 'precision health' OR personalized OR personalised OR tailored OR individualized AND telemedicine OR telehealth OR 'virtual medicine' OR 'virtual health' OR 'mobile health' OR mhealth OR ehealth OR 'electronic health' OR 'digital health' OR 'digital interventions' OR 'mhealth interventions' OR 'electronic interventions' OR 'remote healthcare' OR 'mobile applications' OR smartphone OR wearable* OR "wearable electronic device*" AND hypertension OR "high blood pressure" OR "elevated blood pressure" OR hypertensive |
