# Supplementary material for: Trends and Gaps in Digital Precision Hypertension Management: Scoping Review
Source: J Med Internet Res. 2025 Feb 10;27:e59841. doi: 10.2196/59841 (PMC11851032; doi:10.2196/59841)
Supplement: Multimedia Appendix 3 [file jmir_v27i1e59841_app3.docx]

Multimedia Appendix 3. Characteristics of included studies (N = 46).

| **First Author (year)** | **Country** | **Study Design** | **Precision Health Focus** | **Sample size** | **Digital technology** | **Additional clinical conditions** | **Disciplines involved** |
| --- | --- | --- | --- | --- | --- | --- | --- |
| Abrar et al. (2020) | Malaysia | Prospective observational | Prediction model | 35 | ML algorithm | None | Computer Science and Information Technology |
| Bakre et al. (2022) | US | Retrospective cohort | Phenotyping | 11,934 | Mobile phone Web platform | HLD, DM, Obesity | Medical and Health Sciences |
| Beran et al. (2018) | US | Mixed-methods | Personalized intervention | 450 | BP monitor  Mobile phone | None | Medical and Health Sciences |
| Bernal et al. (2021) | Spain | Proof-of-concept | Prediction model | 7 | Wearable device  Mobile phone  Web platform  ML algorithm | HLD, DM, Obesity | Information and Communication Sciences |
| Bertsimas et al. (2022) | US | Retrospective observational | Prediction model | 19,926 | EHR  ML algorithms | None | School of Management, Mathematical Sciences |
| Blood et al. (2022) | US | Prospective cohort | Personalized intervention | 10803 | BP monitor  Mobile phone | HLD | Medical and Health Sciences |
| Bosworth et al. (2018) | US | RCT | Personalized intervention | 428 | BP monitor  Mobile phone | HLD, DM | Medical and Health Sciences |
| Brewer et al. (2023) | US | Mixed-methods | Personalized intervention | 16 | Mobile phone | HLD, DM, Obesity | Medical and Health Sciences |
| Cano et al. (2021) | Spain | Cross-sectional | Prediction model | 86 | Wearable device  ML algorithm  EHR | None | Electrical Engineering, Medical and Health Sciences |
| Chandler et al. (2019) | US | RCT | Personalized intervention | 56 | Mobile phone  BP monitor  Electronic medication trays/ pill boxes | None | Medical and Health Sciences |
| Chen et al. (2016) | US | Retrospective cohort | Phenotyping | 2,521 | EHR ML algorithm | None | Computational Science and Engineering, Medical and Health Sciences |
| Chiang et al. (2021) | US | RCT | Prediction model  Personalized intervention | 25 | Wearable device  BP monitor  ML algorithm | None | Electrical and Computer Engineering, Medical and Health Sciences |
| Choudhry et al. (2018) | US | RCT | Personalized intervention | 4078 | Mobile phone | HLD, DM | Medical and Health Sciences |
| David et al. (2023) | Brazil | Secondary data analysis of RCT | Personalized intervention | 231 | Mobile phone  BP monitor | DM | Medical and Health Sciences, Informatics |
| Davidson et al. (2015) | US | RCT | Personalized intervention | 38 | Mobile phone  BP monitor  Electronic medication tray | None | Medical and Health Sciences |
| Glynn et al. (2015) | Ireland | Qualitative | Personalized intervention | 50 | Views on technology use (mobile phones, internet, apps) | None | Medical and Health Sciences, Engineering and Informatics |
| Guthrie et al. (2019) | US | Retrospective cohort | Personalized intervention  Prediction model | 172 | Mobile phone  BP monitor  ML algorithm | None | Medical and Health Sciences, Computer Science |
| Hellem et al. (2023a) | US | Qualitative | Personalized intervention | 86 | Mobile phone app | None | Medical and Health Sciences |
| Hellem et al. (2023b) | US | Qualitative | Phenotyping | 13 | Mobile phone  BP monitor | None | Medical and Health Sciences |
| Hu et al. (2023) | US | Retrospective observational | Prediction model | 42,792 | EHR Wearable device ML algorithms | None | Medical and Health Sciences, Electrical and Computer Engineering, Biomedical Engineering |
| Jeong et al. (2018) | South Korea | Pilot RCT | Personalized intervention | 35 | Mobile phone  BP monitor | DM | Medical and Health Sciences |
| Jimeng et al. (2014) | US | Retrospective cohort | Prediction model | 1,294 | EHR  ML algorithms | None | Medical and Health Sciences, Informatics,  Electrical Engineering and Computer Science |
| Kario et al. (2021) | Japan | RCT | Personalized intervention | 390 | Mobile phone  BP monitor | None | Medical and Health Sciences, Biomedical Informatics |
| Kassavou et al. (2020) | England | RCT | Personalized intervention | 135 | Mobile phone | DM2 | Medical and Health Sciences |
| Kelly et al. (2022) | US | Genome sequencing | Phenotyping | 764,135 | Genomic databases | None | Medical and Health Sciences |
| Klein et al. (2020) | US | Prospective cohort | Personalized intervention | 38 | Mobile phone | Bipolar disorder | Medical and Health Sciences |
| Leitner et al. (2022) | US | RCT | Personalized intervention  Prediction model | 38 | Wearable device  Mobile phone  BP monitor  ML algorithm | None | Medical and Health Sciences, Electrical and Computer Engineering |
| Lewinski et al. (2019) | US | Pilot study | Personalized intervention | 118 | Mobile phone | DM2 | Medical and Health Sciences, Biostatistics and Bioinformatics |
| Lv et al. (2017) | US | Pre-post | Personalized intervention | 149 | Mobile phone app  BP monitor  Web-based system  Pedometer | None | Medical and Health Sciences |
| McBride et al. (2020) | Ireland | Qualitative | Personalized intervention | 11 | Mobile phone app | None | Medical and Health Sciences |
| McGillicuddy et al. (2013) | US | Pilot RCT | Personalized intervention | 20 | Mobile phone  Electronic medication tray  BP monitor | Kidney transplant | Medical and Health Sciences |
| Naqvi et al. (2022) | US | Pilot RCT | Personalized intervention | 50 | Electronic tablet  BP monitor | Stroke | Medical and Health Sciences |
| Payne Riches et al. (2021) | United Kingdom | Feasibility RCT | Personalized intervention | 47 | Mobile phone app | None | Medical and Health Sciences |
| Petrella et al. (2014) | Canada | RCT | Personalized intervention | 149 | Mobile phone app  BP monitor  Pedometer | DM2 | Medical and Health Sciences |
| Rodriguez et al. (2015) | US | RCT | Personalized intervention | 544 | Mobile phone | DM2 | Medical and Health Sciences |
| Rodriguez et al. (2019) | US | RCT | Personalized intervention | 533 | Mobile phone | None | Medical and Health Sciences |
| Saleh et al. (2018) | Lebanon | Mixed-methods | Personalized intervention | 606 | Mobile phone | DM2 | Medical and Health Sciences, Health Policy and Management |
| Schoenthaler et al. (2020) | US | Pilot RCT | Personalized intervention | 42 | Electronic tablet | DM2 | Medical and Health Sciences |
| Shea et al. (2022) | US | Pilot RCT | Personalized intervention | 140 | Mobile phone | None | Medical and Health Sciences |
| Steinberg et al. (2020) | US | Feasibility RCT | Personalized intervention | 140 | Mobile phone app | None | Medical and Health Sciences |
| Thiboutot et al. (2013) | US | RCT | Personalized intervention | 500 | Website | DM | Medical and Health Sciences |
| Van Emmenis et al. (2022) | England | Qualitative | Personalized intervention | 20 | Mobile phone app | DM2 | Medical and Health Sciences |
| Wang et al. (2023) | Hong Kong | Pilot RCT | Personalized intervention | 49 | Telemedicine mobile app  BP monitor | None | Medical and Health Sciences |
| Willis et al. (2022) | Sweden | Prospective cohort | Personalized intervention | 7752 | Mobile phone app  BP monitor | None | Medical and Health Sciences, Health Economics |
| Ye et al. (2020) | US | Retrospective analysis | Prediction model | 245,499 | EHR ML algorithms | None | Medical and Health Sciences, Biomedical Informatics |
| Zhang et al. (2022) | China | RCT | Personalized intervention | 192 | Wearable device  Mobile phone | HLD, DM | Medical and Health Sciences |

RCT = Randomized control trial

BP = Blood pressure

ML = Machine learning

AI = Artificial intelligence

EHR = Electronic health records

HLD = Hyperlipidemia

DM = Diabetes mellitus

References

1. Abrar S, Loo CK, Kubota N, Tahir GA, Ieee, editors. A Personalised Blood Pressure Prediction System using Gaussian Mixture Regression and Online Recurrent Extreme Learning Machine. International Symposium on Community-Centric Systems (CcS); 2020 Sep 23-26; Tokyo, JAPAN; 2020.
2. Bakre S, Shea B, Langheier J, Hu EA. Blood Pressure Control in Individuals With Hypertension Who Used a Digital, Personalized Nutrition Platform: Longitudinal Study. Jmir Formative Research. 2022 Mar;6(3). PMID: WOS:000854073700038. doi: 10.2196/35503.
3. Beran M, Asche SE, Bergdall AR, Crabtree B, Green BB, Groen SE, et al. Key components of success in a randomized trial of blood pressure telemonitoring with medication therapy management pharmacists. Journal of the American Pharmacists Association: JAPhA. 2018;58(6):614-21. PMID: 132913573. Language: English. Entry Date: 20190619. Revision Date: 20191103. Publication Type: journal article. doi: 10.1016/j.japh.2018.07.001.
4. Bernal SL, Valverde JM, Celdran AH, Perez GM. SENIOR: An Intelligent Web-Based Ecosystem to Predict High Blood Pressure Adverse Events Using Biomarkers and Environmental Data. Applied Sciences-Basel. 2021 Mar;11(6). PMID: WOS:000645815200001. doi: 10.3390/app11062506.
5. Bertsimas D, Borenstein ARA, Dauvin A, Orfanoudaki A. Ensemble machine learning for personalized antihypertensive treatment. Naval Research Logistics. 2022 /;69(5):669-88. doi: 10.1002/nav.22040.
6. Blood AJ, Cannon CP, Gordon WJ, Mailly C, Maclean T, Subramaniam S, et al. Results of a Remotely Delivered Hypertension and Lipid Program in More Than 10000 Patients Across a Diverse Health Care Network. JAMA Cardiology. 2023;8(1):12-21. doi: 10.1001/jamacardio.2022.4018.
7. Bosworth HB, Olsen MK, McCant F, Stechuchak KM, Danus S, Crowley MJ, et al. Telemedicine cardiovascular risk reduction in veterans: The CITIES trial. American Heart Journal. 2018 May;199:122-9. PMID: WOS:000432183300017. doi: 10.1016/j.ahj.2018.02.002.
8. Brewer LC, Jones C, Slusser JP, Pasha M, Lalika M, Chacon M, et al. mHealth Intervention for Promoting Hypertension Self-management Among African American Patients Receiving Care at a Community Health Center: Formative Evaluation of the FAITH! Hypertension App. Jmir Formative Research. 2023;7. PMID: WOS:001021685100002. doi: 10.2196/45061.
9. Cano J, Hornero F, Quesada A, Martinez-Rodrigo A, Alcaraz R, Rieta JJ, editors. Improved discrimination between healthy and hypertensive individuals combining photoplethysmography and electrocardiography. 2021 Computing in Cardiology (CinC), 13-15 Sept 2021; 2021; Piscataway, NJ, USA: IEEE.
10. Chandler J, Sox L, Kellam K, Feder L, Nemeth L, Treiber F. Impact of a Culturally Tailored mHealth Medication Regimen Self-Management Program upon Blood Pressure among Hypertensive Hispanic Adults. International Journal of Environmental Research and Public Health. 2019 Apr;16(7). PMID: WOS:000465595800139. doi: 10.3390/ijerph16071226.
11. Chen R, Sun J, Dittus RS, Fabbri D, Kirby J, Laffer CL, et al. Patient Stratification Using Electronic Health Records from a Chronic Disease Management Program. IEEE J Biomed Health Inform. 2016 Jan 4. PMID: 26742152. doi: 10.1109/jbhi.2016.2514264.
12. Chiang PH, Wong M, Dey S. Using Wearables and Machine Learning to Enable Personalized Lifestyle Recommendations to Improve Blood Pressure. Ieee Journal of Translational Engineering in Health and Medicine. 2021;9. PMID: WOS:000688263400001. doi: 10.1109/jtehm.2021.3098173.
13. Choudhry NK, Isaac T, Lauffenburger JC, Gopalakrishnan C, Lee M, Vachon A, et al. Effect of a Remotely Delivered Tailored Multicomponent Approach to Enhance Medication Taking for Patients With Hyperlipidemia, Hypertension, and Diabetes The STIC2IT Cluster Randomized Clinical Trial. Jama Internal Medicine. 2018 Sep;178(9):1182-9. PMID: WOS:000443911200011. doi: 10.1001/jamainternmed.2018.3189.
14. David CN, Iochpe C, Harzheim E, Sesin GP, Goncalves MR, Moreira LB, et al. Effect of Mobile Health Interventions on Lifestyle and Anthropometric Characteristics of Uncontrolled Hypertensive Participants: Secondary Analyses of a Randomized Controlled Trial. Healthcare. 2023 Apr;11(8). PMID: WOS:000979443300001. doi: 10.3390/healthcare11081069.
15. Davidson T, Favela A, Villamizar-Escobar J, Brunner-Jackson B, Mueller M, Treiber F. Evaluation of a mobile health enabled medication adherence and blood pressure control program in hispanic uncontrolled hypertensive adults. Psychosomatic Medicine. 2015;77(3):A94-A5.
16. Glynn L, Casey M, Walsh J, Hayes PS, Harte RP, Heaney D. Patients' views and experiences of technology based self-management tools for the treatment of hypertension in the community: A qualitative study. Bmc Family Practice. 2015 Sep;16. PMID: WOS:000360884800001. doi: 10.1186/s12875-015-0333-7.
17. Guthrie NL, Berman MA, Edwards KL, Appelbaum KJ, Dey S, Carpenter J, et al. Achieving Rapid Blood Pressure Control With Digital Therapeutics: Retrospective Cohort and Machine Learning Study. JMIR Cardio. 2019 Mar 12;3(1):e13030. PMID: 31758792. doi: 10.2196/13030.
18. Hellem AK, Casetti A, Bowie K, Golbus JR, Merid B, Nallamothu BK, et al. A Community Participatory Approach to Creating Contextually Tailored mHealth Notifications: myBPmyLife Project. Health Promotion Practice. 2023 2023 Jan. PMID: WOS:000922437100001. doi: 10.1177/15248399221141687.
19. Hellem AK, Whitfield C, Casetti A, Robles MC, Dinh M, Meurer W, et al. Engagement in Self-measured Blood Pressure Monitoring Among Medically Underresourced Participants (the Reach Out Trial): Digital Framework Qualitative Study. JMIR Cardio. 2023 Apr 7;7:e38900. PMID: 37027200. doi: 10.2196/38900.
20. Hu Y, Huerta J, Cordella N, Mishuris RG, Paschalidis IC. Personalized hypertension treatment recommendations by a data-driven model. Bmc Medical Informatics and Decision Making. 2023 Mar;23(1). PMID: WOS:000941964000003. doi: 10.1186/s12911-023-02137-z.
21. Jeong S, Choi H, Gwon SH, Kim J. Telephone Support and Telemonitoring for Low-Income Older Adults. Research in Gerontological Nursing. 2018 Jul-Aug;11(4):198-206. PMID: WOS:000441830300005. doi: 10.3928/19404921-20180502-01.
22. Jimeng S, McNaughton CD, Ping Z, Perer A, Gkoulalas-Divanis A, Denny JC, et al. Predicting changes in hypertension control using electronic health records from a chronic disease management program. Journal of the American Medical Informatics Association. 2014 03/;21(2):337-44. doi: 10.1136/amiajnl-2013-002033.
23. Kario K, Nomura A, Harada N, Okura A, Nakagawa K, Tanigawa T, et al. Efficacy of a digital therapeutics system in the management of essential hypertension: The HERB-DH1 pivotal trial. European Heart Journal. 2021;42(40):4111-22. doi: 10.1093/eurheartj/ehab559.
24. Kassavou A, Mirzaei V, Brimicombe J, Edwards S, Massou E, Prevost AT, et al. A Highly Tailored Text and Voice Messaging Intervention to Improve Medication Adherence in Patients With Either or Both Hypertension and Type 2 Diabetes in a UK Primary Care Setting: Feasibility Randomized Controlled Trial of Clinical Effectiveness. Journal of Medical Internet Research. 2020 May;22(5). PMID: WOS:000533788500001. doi: 10.2196/16629.
25. Kelly TN, Sun X, He KY, Brown MR, Taliun SAG, Hellwege JN, et al. Insights From a Large-Scale Whole-Genome Sequencing Study of Systolic Blood Pressure, Diastolic Blood Pressure, and Hypertension. Hypertension. 2022;79(8):1656-67. doi: 10.1161/HYPERTENSIONAHA.122.19324.
26. Klein P, Aebi ME, Sajatovic M, Depp C, Moore D, Blixen C, et al. Differential Medication Attitudes to Antihypertensive and Mood Stabilizing Agents in response to an Automated Text-Messaging Adherence Enhancement Intervention. J Behav Cogn Ther. 2020 Apr;30(1):57-64. PMID: 33409504. doi: 10.1016/j.jbct.2020.03.015.
27. Leitner J, Chiang PH, Khan B, Dey S, editors. An mHealth Lifestyle Intervention Service for Improving Blood Pressure using Machine Learning and IoMTs. 2022 IEEE International Conference on Digital Health (ICDH), 10-16 July 2022; 2022; Piscataway, NJ, USA: IEEE.
28. Lewinski AA, Patel UD, Diamantidis CJ, Oakes M, Baloch K, Crowley MJ, et al. Addressing Diabetes and Poorly Controlled Hypertension: Pragmatic mHealth Self-Management Intervention. Journal of Medical Internet Research. 2019 Apr;21(4). PMID: WOS:000463859400001. doi: 10.2196/12541.
29. Lv N, Xiao L, Simmons ML, Rosas LG, Chan A, Entwistle M. Personalized Hypertension Management Using Patient-Generated Health Data Integrated With Electronic Health Records (EMPOWER-H): Six-Month Pre-Post Study. Journal of Medical Internet Research. 2017 Sep;19(9). PMID: WOS:000411099600001. doi: 10.2196/jmir.7831.
30. McBride CM, Morrissey EC, Molloy GJ. Patients' Experiences of Using Smartphone Apps to Support Self-Management and Improve Medication Adherence in Hypertension: Qualitative Study. Jmir Mhealth and Uhealth. 2020 Oct;8(10). PMID: WOS:000587430300001. doi: 10.2196/17470.
31. McGillicuddy JW, Gregoski MJ, Weiland AK, Rock RA, Brunner-Jackson BM, Patel SK, et al. Mobile Health Medication Adherence and Blood Pressure Control in Renal Transplant Recipients: A Proof-of-Concept Randomized Controlled Trial. Jmir Research Protocols. 2013 Jul-Dec;2(2). PMID: WOS:000218782100011. doi: 10.2196/resprot.2633.
32. Naqvi IA, Strobino K, Cheung YK, Li HL, Schmitt K, Ferrara S, et al. Telehealth After Stroke Care Pilot Randomized Trial of Home Blood Pressure Telemonitoring in an Underserved Setting. Stroke. 2022 Dec;53(12):3538-47. PMID: WOS:000928051200019. doi: 10.1161/strokeaha.122.041020.
33. Payne Riches S, Piernas C, Aveyard P, Sheppard JP, Rayner M, Albury C, et al. A Mobile Health Salt Reduction Intervention for People With Hypertension: Results of a Feasibility Randomized Controlled Trial. JMIR Mhealth Uhealth. 2021 Oct 21;9(10):e26233. PMID: 34673535. doi: 10.2196/26233.
34. Petrella RJ, Stuckey MI, Shapiro S, Gill DP. Mobile health, exercise and metabolic risk: a randomized controlled trial. Bmc Public Health. 2014 Oct;14. PMID: WOS:000345350400001. doi: 10.1186/1471-2458-14-1082.
35. Rodriguez MA, Friedberg JP, Wang B, Fang Y, Natarajan S. Sustain ability of a tailored behavioral intervention to improve hypertension control: Outcomes of a randomized controlled trial. Journal of General Internal Medicine. 2015;30:S261-S2.
36. Rodriguez MA, Friedberg JP, DiGiovanni A, Wang B, Wylie-Rosett J, Hyoung S, et al. A Tailored Behavioral Intervention to Promote Adherence to the DASH Diet. Am J Health Behav. 2019 Jul 1;43(4):659-70. PMID: 31239010. doi: 10.5993/ajhb.43.4.1.
37. Saleh S, Farah A, El Arnaout N, Dimassi H, El Morr C, Muntaner C, et al. mHealth use for non-communicable diseases care in primary health: patients' perspective from rural settings and refugee camps. Journal of Public Health. 2018 Dec;40:52-63. PMID: WOS:000456088600007. doi: 10.1093/pubmed/fdy172.
38. Schoenthaler A, Leon M, Butler M, Steinhaeuser K, Wardzinski W. Development and Evaluation of a Tailored Mobile Health Intervention to Improve Medication Adherence in Black Patients With Uncontrolled Hypertension and Type 2 Diabetes: Pilot Randomized Feasibility Trial. Jmir Mhealth and Uhealth. 2020 Sep;8(9). PMID: WOS:000591962900001. doi: 10.2196/17135.
39. Shea S, Thompson JLP, Schwartz JE, Chen Y, de Ferrante M, Vanderbeek AM, et al. The Retail Outlet Health Kiosk Hypertension Trial (ROKHYT): Pilot Results. American Journal of Hypertension. 2022;35(1):103-10. doi: 10.1093/ajh/hpab129.
40. Steinberg DM, Kay MC, Svetkey LP, Askew S, Christy J, Burroughs J, et al. Feasibility of a Digital Health Intervention to Improve Diet Quality Among Women With High Blood Pressure: Randomized Controlled Feasibility Trial. Jmir Mhealth and Uhealth. 2020 Dec;8(12). PMID: WOS:000619938400001. doi: 10.2196/17536.
41. Thiboutot J, Sciamanna CN, Falkner B, Kephart DK, Stuckey HL, Adelman AM, et al. Effects of a web-based patient activation intervention to overcome clinical inertia on blood pressure control: cluster randomized controlled trial. Journal of Medical Internet Research. 2013;15(9):e158-e. PMID: 104093604. Language: English. Entry Date: 20140509. Revision Date: 20211029. Publication Type: journal article. doi: 10.2196/jmir.2298.
42. Van Emmenis M, Jamison J, Kassavou A, Hardeman W, Naughton F, A'Court C, et al. Patient and practitioner views on a combined face-to-face and digital intervention to support medication adherence in hypertension: a qualitative study within primary care. BMJ Open. 2022 Feb 28;12(2):e053183. PMID: 35228280. doi: 10.1136/bmjopen-2021-053183.
43. Wang SQ, Leung M, Leung SY, Han JH, Leung W, Hui EL, et al. Safety, Feasibility, and Acceptability of Telemedicine for Hypertension in Primary Care: A Proof-of-concept and Pilot Randomized Controlled Trial (SATE-HT). Journal of Medical Systems. 2023 Mar;47(1). PMID: WOS:000947758000001. doi: 10.1007/s10916-023-01933-4.
44. Willis M, Darwiche G, Carlsson M, Nilsson A, Wohlin J, Lindgren P. Real-world long-term effects on blood pressure and other cardiovascular risk factors for patients in digital therapeutics. Blood Pressure Monitoring. 2023;28(2):86-95. doi: 10.1097/MBP.0000000000000633.
45. Ye XY, Zeng QT, Facelli JC, Brixner DI, Conway M, Bray BE. Predicting Optimal Hypertension Treatment Pathways Using Recurrent Neural Networks. International Journal of Medical Informatics. 2020 Jul;139. PMID: WOS:000569077400006. doi: 10.1016/j.ijmedinf.2020.104122.
46. Zhang Y, Tao Y, Zhong Y, Thompson J, Rahmani J, Bhagavathula AS, et al. Feedback based on health advice via tracing bracelet and smartphone in the management of blood pressure among hypertensive patients: A community-based RCT trial in Chongqing, China. Medicine. 2022 Jul;101(28). PMID: WOS:000825571400063. doi: 10.1097/md.0000000000029346.
